# Adipose tissue gene expression of long non-coding RNAs; MALAT1, TUG1 in obesity: is it associated with metabolic profile and lipid homeostasis-related genes expression?

**DOI:** 10.1186/s13098-020-00544-0

**Published:** 2020-04-29

**Authors:** Reyhane Ebrahimi, Karamollah Toolabi, Naghmeh Jannat Ali Pour, Samaneh Mohassel Azadi, Alireza Bahiraee, Fahimeh Zamani-Garmsiri, Solaleh Emamgholipour

**Affiliations:** 1 Department of Clinical Biochemistry, Faculty of Medicine, Tehran University of Medical Sciences, Tehran, Iran; 2 Students’ Scientific Research Center, Tehran University of Medical Sciences, Tehran, Iran; 3 Department of Surgery, Imam Khomeini Hospital, Tehran University of Medical Sciences, Tehran, Iran

**Keywords:** Long non-coding RNAs (lncRNAs), Obesity, Lipogenesis, Adipogenesis

## Abstract

**Background:**

Recent studies point toward the possible regulatory roles of two lncRNAs; metastasis-associated lung adenocarcinoma transcript 1 (MALAT1) and taurine upregulated gene 1 (TUG1) in the pathogenesis of obesity-related disorders and regulation of lipogenesis and adipogenesis. In an attempt to understand the molecules involved in human obesity pathogenesis, we aimed to evaluate the expression of MALAT1 and TUG1 in visceral adipose tissues (VAT) and subcutaneous adipose tissues (SAT) of obese women, as compared to normal-weight women. The mRNA expression of possible target genes including peroxisome proliferator-activated receptor gamma (PPARγ), PPARγ coactivator-1 alpha (PGC1α), sterol regulatory element-binding protein-1c (SREBP-1c), fatty acid synthase (FAS), and acetyl-CoA carboxylase (ACC) which are involved in adipogenesis and lipogenesis were also examined.

**Methods:**

This study was conducted on 20 obese [body mass index (BMI) ≥ 30 kg/m 2] female participants and 19 normal-weight (BMI < 25 kg/m 2) female participants. Real-time PCR was performed to investigate the mRNA expression of the above-mentioned genes in VAT and SAT from all participants.

**Results:**

The results showed lower mRNA levels of TUG1 in both the VAT and SAT of obese women, compared to normal-weight women. Furthermore, TUG1 expression in SAT positively correlated with BMI, waist circumference (WC), hip circumference, HOMA-IR, and insulin levels, eGFR value, creatinine levels, and hs-CRP in all participants independent of age and HOMA-IR. However, VAT mRNA expression of TUG1 had a positive correlation with obesity indices and HOMA-IR and insulin levels in the whole population. Moreover, SAT mRNA level of TUG1 was positively correlated with SAT gene expression of PGC1α, SREBP-1c, FAS, and ACC independent of age and HOMA-IR. Although mRNA expression of MALAT1 did not differ between two groups for any tissue, it was positively correlated with SAT mRNA levels of SREBP-1c, PPARγ, and their targets; FAS and ACC, as well as with VAT mRNA levels of PGC1α.

**Conclusions:**

It seems likely that TUG1 with distinct expression pattern in VAT and SAT are involved in the regulation of lipogenic and adipogenic genes and obesity-related parameters. However, more studies are necessary to establish this concept.

## Background

Obesity is a strong risk factor in various diseases, such as cardiovascular disorders, diabetes, metabolic syndrome, hypertension, and cancer [1]. This global epidemic is increasingly growing in both developed and developing countries, with negative outcomes including increased morbidity [2]. Obesity is strongly correlated with an imbalance between energy intake and energy consumption. It is generally accepted that adipose tissue undergoes a continuous process which affects systemic metabolism and inflammatory status in conditions of excess energy [3, 4]. Specifically, subcutaneous adipose tissue (SAT) and visceral adipose tissue (VAT) as two types of white adipose tissue have distinct cellular, molecular, and clinical characteristics [5]. Therefore, assessment of these two tissues in obesity-related studies is of great importance in order to obtain the information about control of these disorders.

The possible role of long non-coding RNAs in the field of obesity-related research has recently come into focus [2]. LncRNAs are transcripts with lengths greater than 200 nucleotides which are incapable of encoding proteins [6]. These RNAs have emerged as a major regulator of gene expression through epigenetic changes in many processes, including X chromatin inactivation [7], regulating the function of key metabolic genes, cell cycle control, and cell differentiation [8]. Moreover, there is fresh evidence which demonstrates the dysregulation of lncRNAs in different human diseases [9]. Today, the possible role of lncRNAs in the regulatory network of lipid metabolism, the regulation of adipogenesis, adipocyte metabolism, hepatic lipid metabolism, and fat accumulation [2, 10, 11] is being considered. For instance, diet-induced obesity in mice lacking lncRNA (lncOb) caused an increase in fat mass [12]. In another study, the knockdown of adipocyte-specific metabolic-related lncRNAs (ASMERs) in differentiated human adipocytes showed that ASMERs may be possible modulators of adipogenesis, lipid mobilization, and adiponectin expression [13]. Integrated analysis of LncRNA profiles in obese children revealed that hub lncRNA RP11-20G13.3 suppressed adipogenesis [14].

Lipogenesis is a process of converting excess energy into fatty acids, and adipogenesis is defined as developing preadipocytes into mature adipocytes. Both are highly regulated by a transcriptional cascade that mostly includes peroxisome proliferator-activated receptor gamma (PPARγ) and sterol regulatory element-binding protein-1 (SREBP-1). PPARγ, as a nuclear receptor, is linked to lipid synthesis, fat accumulation, and also insulin sensitivity, which is activated by PPARγ coactivator-1 alpha (PGC1α). Likewise, SREBP-1 contributes to cholesterol homeostasis and lipid synthesis in response to carbohydrates [15–17]. These molecules in cooperation with each other, regulate lipogenesis and adipogenesis through inducing fatty acid synthase (FAS) and acetyl-CoA carboxylase (ACC) as two major biosynthetic enzymes for lipid synthesis and adipocyte differentiation [18–20].

Although the role of lncRNAs in the regulation of fat accumulation, lipogenesis, and adipogenesis is increasingly being explored, the current understanding of the function of these molecules in the initiation and development of obesity in humans is still in its infancy.

More recently, great attention has been paid to the probable role of two novel lncRNAs; metastasis-associated lung adenocarcinoma transcript 1 (MALAT1) and taurine upregulated gene 1 (TUG1) in the pathogenesis of metabolic disorders. MALAT1 is a highly conserved lncRNA with an extent of 8.7-kb. Its name is derived from the first cells within which it was discovered. The role of MALAT1 is mostly seen in the nucleus, having a primary effect on gene transcription (in collaboration with other regulators) [21, 22]. There is evidence that *Malat1* expression is down-regulated in subcutaneous adipose tissue from obese animal models [23], while a high level of MALAT1 was seen in secretome from the omental depot of obese adipose-derived stem cells [24]. There is also evidence that serum levels of MALAT1 in patients with diabetes who smoked were significantly higher in comparison with nonsmokers patients [25]. TUG1 is also a highly conserved lncRNA with an extent of 6.7-kb, and was firstly identified after taurine treatment, along with its up-regulation function [26, 27]. It was observed that TUG1 expression is decreased in a non-obese diabetic animal model and its downregulation is associated with diabetes [9]. Overexpression of TUG1 alleviates extracellular matrix accumulation, including TGF-β1, collagen IV, and fibronectin in diabetic nephropathy through a mechanism dependent on PPARγ [28]. Moreover, animal studies and in vitro experiments point toward the possible regulatory roles of MALAT1 and TUG1 in regulating the master pathways of energy hemostasis, including PPARγ, PGC1α, SREBP-1, FAS, and ACC [29, 30].

To the best of the authors’ knowledge, no data were available on the alteration in the expression levels of MALAT1 and TUG1 in human adipose tissue and their possible association with metabolic parameters in the context of obesity. Therefore, in an attempt to realize the molecules involved in the pathogenesis of obesity, here, we aimed to evaluate the expression of MALAT1 and TUG1, as well as plausible target genes (PPARγ, PGC1α, SREBP-1c, FAS, and ACC), in VAT and SAT from obese women compared to normal-weight subjects. The study also aimed to examine the possible association of TUG1 and MALAT1 gene expression with biochemical and clinical indices in obese patients.

## Methods and materials

### Study population

This case–control study was performed on twenty obese patients (BMI ≥ 30 kg/m^2^), and nineteen normal-weight subjects (BMI ≤ 25 kg/m^2^). All subjects were women and aged between 20 and 53 years. The obese patients were selected from women who qualified for bariatric surgery (vertical sleeve gastrectomy and Roux-en-Y gastric bypass) under specialist supervision at the bariatric surgery center of Erfan hospital. The normal-weight controls were recruited from women who underwent elective cholecystectomy or inguinal hernia at the center of advanced laparoscopic surgeries at Sina and Loqman Hakim hospitals, Tehran, Iran. It should be noted that all participants were of Iranian ethnic.

Subjects were excluded if they had type 2 diabetes, acute and chronic infectious diseases, renal disease, liver disease, autoimmune disease, cardiovascular disease, cancer, hormonal and thyroid dysfunctions, pregnancy, and operation or hospital admission history in the last 6 months. The participants were neither smoking at the time of study nor taking drugs known to alter metabolism (e.g. weight loss control drugs, metformin, statin medications, hypoglycemic and hypolipidemic drugs). However, it should be noted that only two patients in the obese group were receiving antihypertensive drugs.

More importantly, none of the subjects were post-menopause at the time of inclusion in the study. The study protocol was in accordance with the Declaration of Helsinki and was approved by the ethics committee of Tehran University of Medical Sciences (IR.TUMS.VCR.REC.1397.827). Written informed consent was obtained from each subject before surgery. The anthropometric indices of all subjects were assessed, including age, weight, height, waist circumference (WC), BMI, hip circumference, waist-to-hip-ratio (WHR), and blood pressure. BMI was measured based on the ratio of weight in kg divided by height in m^2^ to assess the fatness of participants. WC was calculated at the midpoint between the lowest rib and the iliac crest using a flexible inch tape. Moreover, hip was measured at the maximum circumference of the buttocks. WHR was measured based on the ratio of WC in cm divided by hip circumference in cm. A manual sphygmomanometer was used for determining the systolic and diastolic blood pressures of subjects after 15 min resting in a sitting position.

### Biochemical and laboratory measurements

Blood samples were obtained from all subjects after an overnight fast and before surgery. The blood was collected by venipuncture from an antecubital vein into sterile BD Vacutainer tubes. The serum was separated and immediately frozen at − 80 °C until the following analyses.

Fasting blood glucose (FBG), uric acid, urea, creatinine, low-density lipoprotein cholesterol (HDL-C), low-density lipoprotein cholesterol (LDL-C), triglyceride (TG), total cholesterol (TC), alkaline phosphatase (ALP), aspartate aminotransferase (AST), and alanine aminotransferase (ALT) were measured by auto analyzer using commercial kits (Pars Azmoon, Tehran, Iran). Furthermore, high sensitivity-reactive protein (hs-CRP) was assessed by an immunoturbidometric method using the Roche Integra analyzer. The fasting plasma insulin was quantified using ECL method in the Cobas6000 E601 auto analyzer. To examine the insulin resistance (IR), homeostasis model assessment of IR (HOMA-IR) was estimated with the following equation: fasting blood glucose (mg/dL) × fasting blood insulin (µU/mL)/405.

Estimated glomerular filtration rate (eGFR) as the best index of kidney function was calculated based on the abbreviated MDRD equation [31]:

$$eGFR = 186 \times \left( {serum \,creatinine } \right)^{ - 1.154} \times \left( {age} \right)^{ - 0.203} \times 1.212\left( {if\,black} \right) \times 0.742\left( {if \,female} \right).$$

### Adipose tissue samples

VAT and SAT were collected during the surgical procedure from the obese subjects and the normal-weight controls. In brief, 0.5–1 grams of visceral fat derived from the omentum was eviscerated by a specialist surgeon during the bariatric or elective surgery. Moreover, by cutting a small aperture under the skin, subcutaneous fat (approximately 0.5 g) was collected with a scalpel. Biopsy samples were washed in sterile and cold phosphate-buffered saline and immediately frozen in liquid nitrogen. Then, the adipose tissue samples were stored at − 80 °C until the subsequent experiment.

### Real-time quantitative polymerase chain reaction (PCR)

Firstly, frozen biopsy samples were homogenized in liquid nitrogen. Total RNA was extracted from frozen VAT and SAT using the RNeasy Lipid Tissue Mini Kit (Qiagen GmbH, Germany). RNA purity and integrity were evaluated by determining the ratio of absorbance at 260 nm to that at 280 nm and agarose gel electrophoresis, respectively. The first-strand complementary DNA (cDNA) synthesis was performed on 1000 ng of DNase-treated RNA using the PrimeScript 1st Strand cDNA Synthesis kit (Takara, Japan).

Real-time qPCR was performed based on the criteria of the MIQE guidelines [32]. Quantitative Real-time PCR was accomplished using BioFACT™ 2X Real-Time PCR Master Mix (For SYBR Green I) in a Step-One-Plus TM real-time (ABI Applied Biosystems). In detail, the conditions for PCR amplification reactions were as follows: an initial denaturation step at 95 °C for 15 min followed by 40 cycles of amplification encompassing denaturation at 95 °C for 20 s, annealing at 60 °C for 45 s. Following this reaction, a melting curve analysis was generated by increasing the temperature from 65 to 95 °C with a continuous collection of the SYBR Green fluorescence signal. The curves in the dissociation analyses generated single peaks (unique Tm). We used β-actin and glyceraldehyde 3-phosphate dehydrogenase (GAPDH) as the reference genes. Since the differences of β-actin and GAPDH expressions were not statistically significant between the obese and non-obese groups, each sample was normalized to the corresponding value of the geometric mean of these reference genes. For each sample, the difference in Ct values (ΔCt) between the target gene and the reference gene (geometric mean of these internal genes) was calculated. We would like to stress that none of the samples had Ct values more than 40. More importantly, efficiency of the amplification for all target genes and reference genes was checked by means of calibration curves. The efficiency (E) of amplification was similar for all target genes and reference genes and ranged from 95% to 100% in all assays. Hence, 2^−∆∆Ct^ method was applied to perform relative quantification. All data were expressed as an n-fold difference relative to the calibrator sample (a mixture of the SAT and VAT tissues). Additional file 1: Table S1 lists the used primer sequences for analyzing the gene expression of MALAT1 and TUG1 and their target genes including PPARγ, PGC1α, SREBP-1c, FAS, and ACC.

### Statistical analysis

The sample size was calculated according to data from the literature about gene expression of TUG1 in subjects with diabetes and without diabetes [30]. In detail, the number of subjects in each group was estimated to be 20 to achieve a difference of 35% in the mean value of TUG1 transcript levels between the studied groups with a confidence level of 95% and a statistical power of 80%.

Data normality was checked by the Shapiro–Wilk test. Laboratory and anthropometric parameters with normal distribution were presented as mean ± standard deviation (SD), and variables without normal distribution were presented as median (interquartile ranges). All values in the figures were shown as the mean ± standard error of mean (SEM).Log-transformation was employed for variables with non-normal distribution. The comparison of gene expression levels, as well as anthropometric and biochemical data between obese patients and normal-weight subjects, was carried out by independent students t-test on log-transformed variables.

Comparison of mRNA levels of all studied genes between the VAT and SAT was done by the paired t-test on log-transformed variables. Subsequently, ANCOVA analysis was performed to remove the effects of potential confounders. Correlation coefficients were calculated using the two-tailed Pearson’s correlation analysis. It should be noted that non-normally distributed variables were log-transformed to generate a normal distribution before further analyses. Pearson partial correlation coefficients were performed to examine the correlation of transcript levels of TUG1 with anthropometric indices, clinical characteristics, and gene expression of possible target genes after controlling for BMI. General linear regression analysis was performed to identify associations of TUG1 and MALAT1 in VAT and SAT of the whole study population with anthropometric indices, clinical characteristics, and gene expression of possible target genes by adjusting for age, and HOMA-IR. A stepwise multivariable linear regression analysis was performed to ascertain the best set of predictors for TUG1 gene expression.

All statistical assessments were two-tailed and P value < 0.05 was considered statistically significant. All data analysis was performed using SPSS 20 (SPSS, Chicago, IL, USA).

## Results

### Laboratory and anthropometric parameters

The metabolic, clinical, and anthropometric parameters of the obese and normal-weight women are summarized in Table 1. Subjects in the obese and non-obese group had a mean age of 34.25 ± 5.81 years and 38.84 ± 9.14 years, respectively (P = 0.07). Obese women had higher obesity indices including BMI, WC, and hip circumference in comparison with the normal-weight subjects, however, no difference in WHR was identified between two groups. Obese women had also a significantly higher serum insulin levels, HOMA-IR values compared with the normal-weight ones. Moreover,eGFR values, serum levels of hs-CRP, LDL-C, total cholestrol, triglycerides, uric acid, total protein, albumin, urea, and creatinine were significantly different between the two groups. However, the serum level of FBG, ALP, AST, and ALT was not statistically different between the two groups.

**Table 1:** The anthropometric, clinical, and metabolic characterizations of the all participants

Characteristics	Normal-weight subjects (n = 19)	Obese subjects (n = 20)	Total difference p value
Age, years	38.84 ± 9.14	34.25 ± 5.81	0.073
BMI, kg/m2	23.49 (22.86–24.34)	41.73 (36.35–46.77)	0.000
WC, cm	85 (83–87)	114 (111.25–120)	0.000
Hip, cm	95 (90–97)	128 (120–133.5)	0.000
WHR, -	0.89 ± 0.04	0.92 ± 0.05	0.068
SBP, mmHg	117.05 ± 11.59	119.25 ± 14.26	0.602
DBP, mmHg	80 (70–80)	76.5 (62.5–87.5)	0.971
FBG, mg/dL	84.45 ± 7.36	86.42 ± 8.33	0.44
Urea nitrogen, mg/dL	23.08 ± 7.78	25.62 ± 5.85	0.256
Creatinine, mg/dL	0.57 ± 0.163	0.73 ± 0.10	0.001
Uric acid, mg/dL	4.02 ± 0.72	5.43 ± 1.02	0.000
eGFR, mL/min/1.73 m^2^	138.31 ± 48.07	99.73 ± 17.57	0.002
HDL-C, mg/dL	43.81 ± 6.34	45.10 ± 7.25	0.557
LDL-C, mg/dL	88.43 ± 30.82	113.05 ± 20.67	0.006
TC, mg/dL	146.51 ± 39.09	179.65 ± 26.58	0.004
TG, mg/dL	93.1 (56.6–127.7)	91.55 (59.87–123.25)	0.786
VLDL-C, mg/dL	21 (15–28)	20 (17–28)	0.679
LDL-C/HDL-C,-	2.076 ± 0.79	2.5 ± 0.60	0.041
AST, U/L	16.7 (12.5–21.4)	21.1 (16.2–23.95)	0.223
ALT, U/L	12.9 (11–22.2)	21.65 (15.35–30.2)	0.406
ALP, U/L	70.29 ± 26.73	72.88 ± 17.09	0.719
Albumin, g/dL	3.7 (3.19–3.92)	4.31 (4.2–4.43)	0.000
TP, g/dL	5.81 ± 0.83	6.81 ± 0.74	0.000
hs-CRP, mg/L	1.8 (1.01–2.5)	4.85 (2.9–10.29)	0.000
HOMA-IR, -	1.69 (0.93–2.24)	3.85 (3.37–5.21)	0.000
Insulin, µU/mL	8.1 (4.63–9.93)	19.45 (15.5–23.41)	0.000

Continuous variables with normal and non-normal distribution were described as the mean ± SD and median (IQR), respectively

BMI, body mass index; WC, waist circumference, WHR, waist-to-hip ratio; SBP, systolic blood pressure; DBP, diastolic blood pressure; FBG, fasting blood glucose; HDL-high-density lipoprotein cholesterol; LDL-C, low-density lipoprotein cholesterol; TC, total cholesterol; TG, triglycerides; AST, aspartate aminotransferase; ALT, alanine aminotransferase; ALP, alkaline phosphatase; TP, total protein; hs-CRP, high-sensitivity C-reactive Protein; HOMA-IR, homeostasis model assessment of insulin resistance; eGFR, estimated glomerular filtration rate

### Expression of MALAT1 and TUG1 in the obese and normal-weight women

**Fig. 1:**
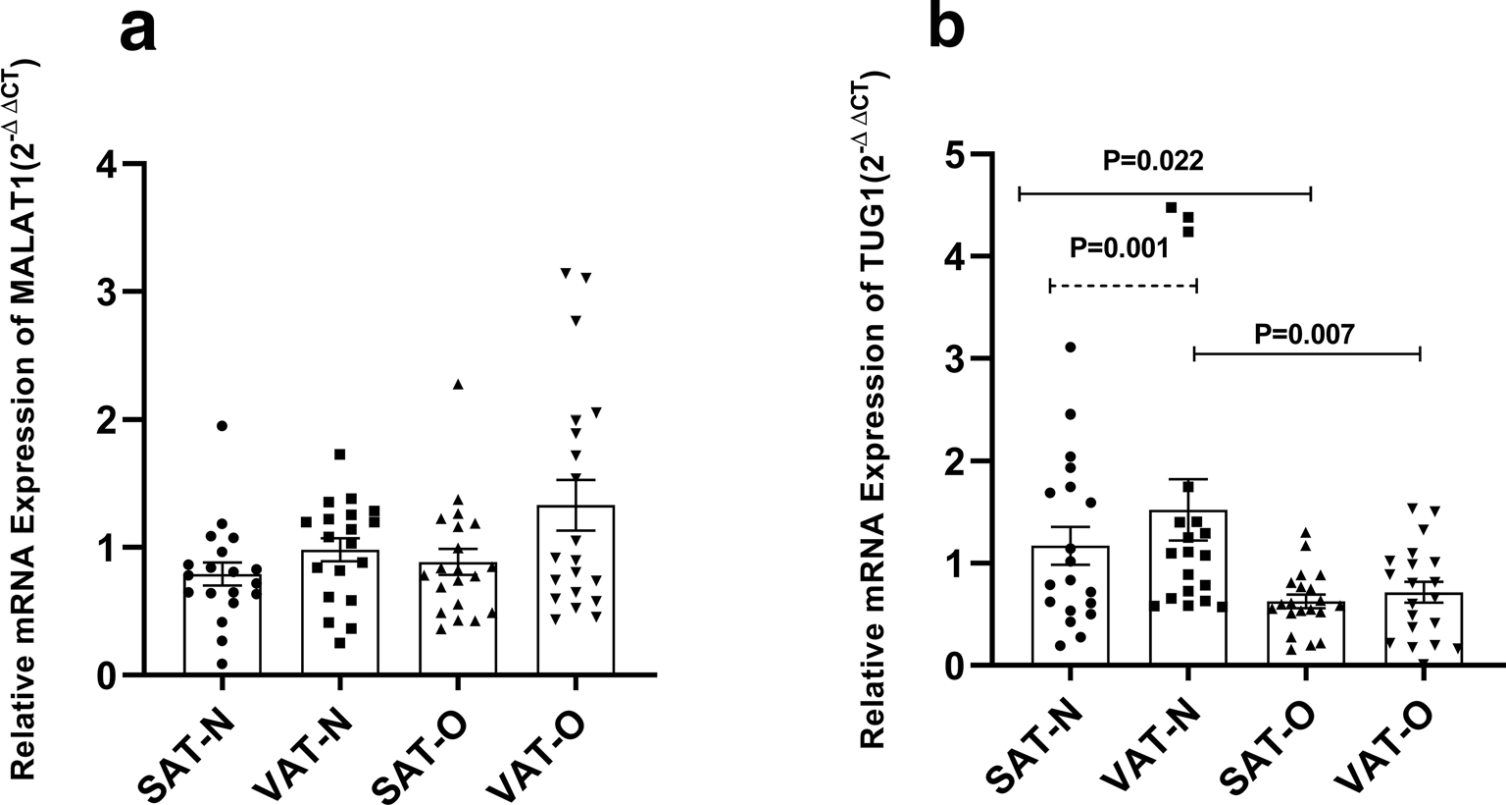
Gene expression of MALAT1 (**a**) and TUG1 (**b**) in the visceral (VAT) and subcutaneous (SAT) adipose tissues of obese (O) subjects (n = 20) and normal-weight (N) individuals (n = 19). All data were expressed as an n-fold difference relative to the calibrator sample (a mixture of the SAT and VAT tissues). Results were shown as the mean ± standard error of mean (SEM)

The gene expression of MALAT1 and TUG1 in VAT and SAT of obese patients (n = 20) and normal-weight women (n = 19) are demonstrated in Fig. 1.

In detail, MALAT1 gene expression in SAT and VAT was not significantly different between the two groups (Fig. 1a). However, TUG1 gene expression was markedly lower in VAT (P = 0.007) and SAT (P = 0.022) of obese participants rather than those found in normal-weight controls (Fig. 1b).

ANCOVA was performed to remove the effect of age on the gene expression of TUG1 and MALAT1 in the VAT and SAT. The results indicated that the decrease in the expression of TUG1 was independent of age both in VAT (P = 0.022) and SAT (P = 0.04) of the obese group. But, no significant difference was revealed in mRNA levels of MALAT-1 in VAT and SAT deposits between the two groups after controlling for age.

Creatinine serum level and HOMA-IR value were statistically different between the two groups. Hence, ANCOVA was performed to adjust the difference in MALAT1 and TUG1 for potential covariates including age, creatinine levels, and HOMA-IR value. The results showed that TUG1 gene expression in SAT of the obese group remained significantly different from that of the normal-weight group after adjustment for afore-mentioned covariates (P = 0.003). However, the difference in TUG1 (P = 0.074) in VAT was not statistically significant between the two groups after controlling for age, HOMA-IR, and creatinine.

When the gene expression pattern of MALAT1 and TUG1 was compared between VAT and SAT in each studied group, a significantly lower TUG1 gene expression in VAT, compared to SAT, was noticed in the normal-weight group (P = 0.001). However, the transcript level of MALAT1 was similar between VAT and SAT in each studied group.

### Expression of PPARγ, PGC1α, SREBP-1c, and their targets; FAS and ACC in the obese and normal-weight women

**Fig. 2:**
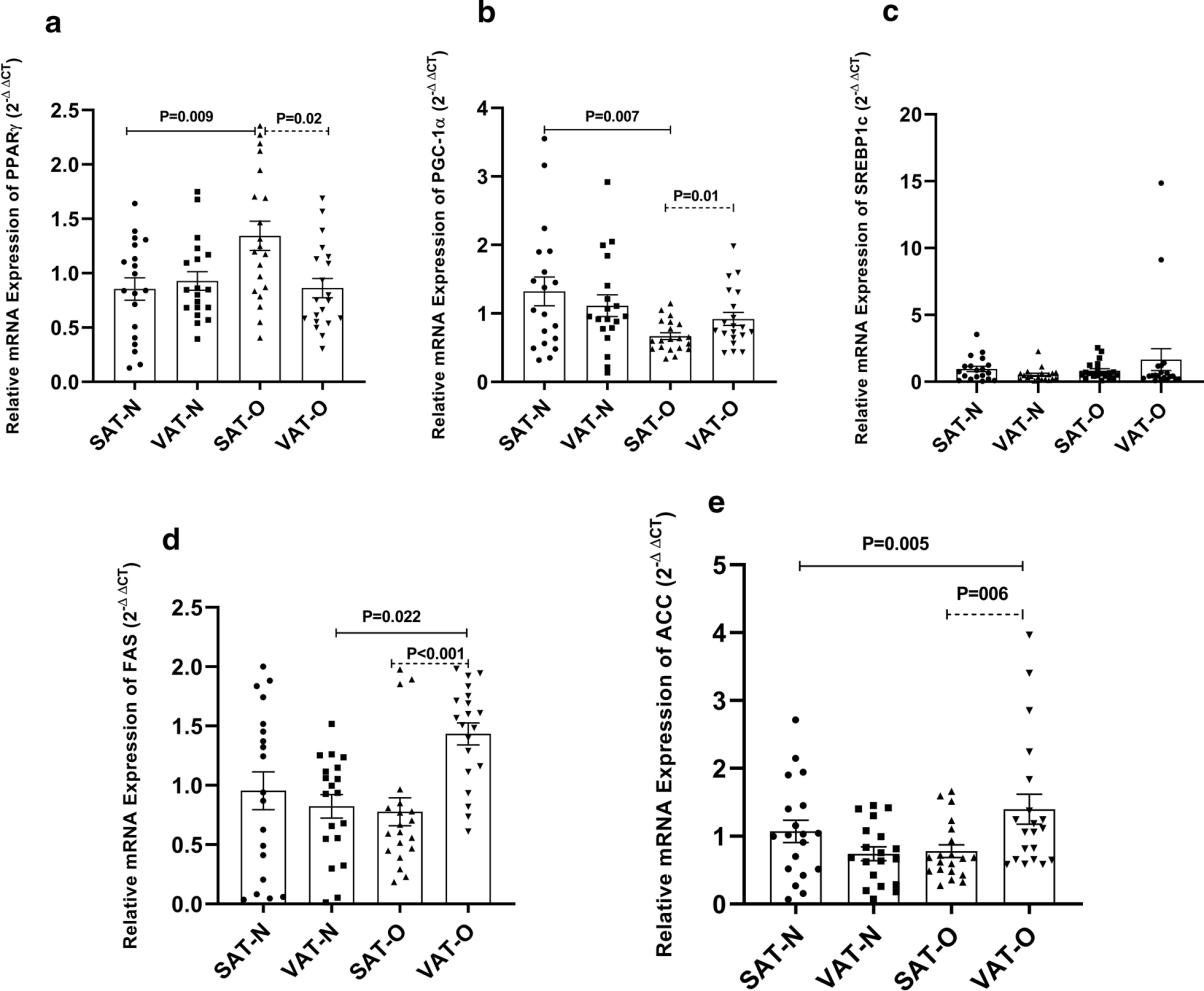
Gene expression of PPARγ (**a**), PGC1α (**b**), SREBP-1c (**c**), FAS (**d**), and ACC (**e**) genes in the visceral (VAT) and subcutaneous (SAT) adipose tissues of obese (O) subjects (n = 20) and normal-weight (N) individuals (n = 19). All data were expressed as an n-fold difference relative to the calibrator sample (a mixture of the SAT and VAT tissues). Results were shown as the mean ± standard error of mean (SEM)

We analyzed the gene expression of PPARγ, PGC1α, SREBP-1c, FAS, and ACC as the main target genes of lipogenesis and adipogenesis in SAT and VAT of obese (n = 20) and normal-weight (n = 19) women (Fig. 2). In SAT of the obese group, the gene expression of PPARγ and PGC1α was significantly increased (P = 0.009) and decreased (P = 0.007) compared to those in SAT of the normal-weight group, respectively. However, PPARγ and PGC1α gene expression in VAT was not significantly different between the two groups (Fig. 2a, b). In addition, FAS (P = 0.022) and ACC (P = 0.005) expression levels were significantly higher in the VAT from obese subjects, in comparison to the normal weight ones. However, no significant difference was revealed in the transcript levels of SREBP-1c in VAT and SAT deposits between the two groups (Fig. 2c). Our data indicated that gene expression of FAS and ACC in SAT was not significantly different between non-obese and obese groups (Fig. 2d, e).

Statistical analysis using ANCOVA test showed that PPARγ (P = 0.020) and PGC1α (P = 0.007) transcript levels remain significantly different from those of the normal-weight group after controlling for age. Furthermore, the results of ANCOVA analysis showed that the increase in gene expression of FAS (P = 0.012) and ACC (P = 0.009) in the VAT from obese women was independent of age.

When we compared the gene expression pattern between VAT and SAT in each study group, we noticed a tendency to higher gene expression in the VAT rather than in the SAT for PGC1α (P = 0.01), FAS (p < 0.001), and ACC (P = 0.006) in the obese group. While PPARγ gene expression (P = 0.02) tended to be higher in the SAT from obese patients.

### Correlation of MALAT1 and TUG1 Expression with Anthropometric and Biochemical Measurements

Bivariate correlation analysis of MALAT1 and TUG1 expression with anthropometric and laboratory parameters in the whole population study is summarized in Tables 2 and 3. The results indicated a significant negative correlation between TUG1 mRNA expression in VAT and BMI (r = − 0.404; P = 0.011), WC (r = − 0.383; P = 0.016), and hip circumference (r = − 0.433; P = 0.006),HOMA-IR (r = − 0.337; P = 0.036) and insulin levels(r = − 0.344; P = 0.032). Moreover, TUG1 mRNA expression in SAT had an inverse correlation with BMI (r = − 0.332; P = 0.039), WC (r = − 0.378; P = 0.018), and hip circumference (r = − 0.425; P = 0.007), creatinine levels (r = − 0.465; P = 0.003) and hs-CRP levels (r = − 0.355; P = 0.027). Moreover, TUG1 transcript level in SAT had a positive correlation with eGFR values (r = 0.421; P = 0.008). Then, all correlations were adjusted for age and HOMA-IR. The results showed that all significant correlations between TUG1 transcript levels in SAT and anthropometric and laboratory parameters remained significant after adjustment for age and HOMA-IR. However, we found no significant correlation between TUG1 gene expression in VAT and obesity indices after controlling for age and HOMA-IR (Table 2).

**Table 2:** Unadjusted and adjusted correlation of TUG1 gene expression in SAT and VAT of the whole population study with anthropometric and metabolic profiles

Characteristics	lncRNA TUG1 in SAT		lncRNA TUG1 in VAT	
	Whole population		Whole population	
	Unadjusted Pearson coefficient	Adjusted for age and HOMA-IR Pearson coefficient	Unadjusted Pearson coefficient	Adjusted for age and HOMA-IR Pearson coefficient
BMI, kg/m^2^	− 0.332^a^	− 0.535^a^	− 0.404^a^	− 0.322
WC, cm	− 0.378^a^	− 0.594^b^	− 0.383^a^	− 0.275
Hip, cm	− 0.425^b^	− 0.732^b^	− 0.433^b^	− 0.340
WHR, -	0.014	0.116	0.026	0.097
FBG, mg/dL	− 0.164	− 0.138	− 0.016	0.068
Creatinine, mg/dL	− 0.465^b^	− 0.577^b^	0.015	0.295
eGFR, mL/min/1.73 m^2^	0.421^b^	0.458^b^	0.029	0.197
HDL-C, mg/dL	0.256	0.247	− 0.207	− 0.215
LDL-C, mg/dL	− 0.020	− 0.027	− 0.239	0.022
TC, mg/dL	− 0.096	− 0.112	− 0.239	0.045
TG, mg/dL	− 0.024	− 0.103	− 0.071	− 0.084
hs-CRP, mg/L	− 0.355^a^	− 0.472^a^	− 0.084	0.232
HOMA-IR, -	− 0.122	–	− 0.337^a^	–
Insulin, µU/mL	− 0.103	–	− 0.344^a^	–

BMI, body mass index; WC, waist circumference; WHR, waist-to-hip ratio; FBG, fasting blood glucose; HDL-high-density lipoprotein cholesterol; LDL-C, low-density lipoprotein cholesterol; TC, total cholesterol; TG, triglycerides; hs-CRP, high-sensitivity C-reactive Protein; HOMA-IR, homeostasis model assessment of insulin resistance; eGFR, estimated glomerular filtration rate

^a^ Correlation is significant at the 0.05 level (2-tailed)

^b^ Correlation is significant at the 0.01 level (2-tailed)

**Table 3:** Unadjusted and adjusted correlation of MALAT1 gene expression in SAT and VAT of the whole population study with anthropometric and metabolic profiles

Characteristics	lncRNA MALAT1 in SAT		lncRNA MALAT1 in VAT	
	Whole population		Whole population	
	Unadjusted Pearson coefficient	Adjusted for age and HOMA-IR Pearson coefficient	Unadjusted Pearson coefficient	Adjusted for age and HOMA-IR Pearson coefficient
BMI, kg/m^2^	0.149	− 0.187	0.210	0.358
WC, cm	0.062	− 0.332	0.180	0.278
Hip, cm	0.133	− 0.213	0.237	0.347
WHR, -	− 0.182	− 0.264	− 0.113	− 0.072
FBG, mg/dL	0.273	0.210	0.091	0.090
Creatinine, mg/dL	0.107	0.102	− 0.023	− 0.074
eGFR, mL/min/1.73 m^2^	− 0.094	0.096	0.047	0.092
HDL-C, mg/dL	− 0.157	− 0.149	0.233	0.229
LDL-C, mg/dL	0.264	0.068	0.167	0.159
TC, mg/dL	0.166	− 0.082	0.238	0.258
TG, mg/dL	0.041	0.069	0.100	0.054
hs-CRP, mg/L	0.205	0.004	− 0.103	− 0.208
HOMA-IR, -	0.319^a^	–	0.046	–
Insulin, µU/mL	0.288	–	0.032	–

BMI, body mass index; WC, waist circumference; WHR, waist-to-hip ratio; FBG, fasting blood glucose; HDL-high-density lipoprotein cholesterol; LDL-C, low-density lipoprotein cholesterol; TC, total cholesterol; TG, triglycerides; hs-CRP, high-sensitivity C-reactive Protein; HOMA-IR, homeostasis model assessment of insulin resistance; eGFR, estimated glomerular filtration rate

^a^ Correlation is significant at the 0.05 level (2-tailed)

We also calculated Pearson partial correlation coefficients to examine the correlation of transcript levels of TUG1 with anthropometric indices and clinical characteristics, after controlling for BMI.

In the whole population, only hsCRP (r = 0.315; P = 0.045) retained significant partial correlation with VAT transcript level of TUG among the variables shown in Table 2 after being adjusted for BMI. After being adjusted for BMI, only hip circumference (r = − 0.311; P = 0.04), eGFR value(r = 0.377; P = 0.02) and creatinine levels (r = − 0.368; P = 0.023) showed significant partial correlation with TUG1 gene expression in SAT of all participants.

A stepwise multivariable linear regression analysis was performed to ascertain the best set of predictors for TUG1 gene expression. Our results showed that creatinine (β = − 0.465, P = 0.003) remains as an independent determinant of TUG1 transcript levels in SAT of all participants.

However, multivariate stepwise linear regression analysis showed that BMI (β = − 0.404, P = 0.011) remains as an independent determinant of TUG1 gene expression in VAT of all participants.

Correlation analysis revealed that MALAT1 mRNA in SAT positively correlated with HOMA-IR (r = − 0.319; P = 0.048) (Table 3).

### Correlation of MALAT1 and TUG1 expression with PPARγ, PGC1α, SREBP-1c, FAS, and ACC gene expression

We also investigated the correlation of MALAT1 and TUG1 mRNA levels with each other and with PPARγ, PGC1α, SREBP-1c, FAS, and ACC gene expressions in SAT and VAT of the whole population study (Tables 4 and 5). According to our findings, there was a positive correlation between the mRNA expression of TUG1 and PGC1α (r = 0.466; P = 0.003), SREBP-1c (r = 0.407; P = 0.01), FAS (r = 0.342; P = 0.033), and ACC (r = 0.415; P = 0.009) in the SAT of the all participants (Table 4). Then, all correlations were adjusted for age and HOMA-IR. The results showed that all significant correlations between TUG1 transcript levels in SAT and PGC1α, SREBP-1c, FAS, and ACC remained significant after adjustment for age and HOMA-IR. Moreover, we found a positive correlation between MALAT1 and TUG1 mRNA levels in SAT after adjustment (Table 4).

**Table 4:** Unadjusted and adjusted correlation of TUG1 gene expression in SAT and VAT of the whole population study with energy homeostasis-related genes

Characteristics	lncRNA TUG1 in SAT		lncRNA TUG1 in VAT	
	Whole population		Whole population	
	Unadjusted Pearson coefficient	Adjusted for age and HOMA-IR Pearson coefficient	Unadjusted Pearson coefficient	Adjusted for age and HOMA-IR Pearson coefficient
PPARγ	− 0.0196	− 0.119	0.090	0.021
PGC1α	0.466^b^	0.497^b^	0.155	0.191
SREBP	0.407^a^	0.465^b^	− 0.059	− 0.041
FAS	0.342^a^	0.378^a^	− 0.093	0.007
ACC	0.415^b^	0.411^b^	− .0143	− 0.049
LncRNA MALAT1	0.299	0.346^a^	− 0.380^a^	− 0.324^a^

SAT, subcutaneous adipose tissue; VAT, visceral adipose tissue; MALAT1, metastasis-associated lung adenocarcinoma transcript 1; TUG1, taurine upregulated gene 1; PPARγ, peroxisome proliferator-activated receptor gamma; PGC1α, PPARγ coactivator-1 alpha; SREBP-1, sterol regulatory element-binding protein α; FAS, fatty acid synthase; ACC, acetyl-CoA carboxylase; HOMA-IR, homeostasis model assessment of insulin resistance

^a^ Correlation is significant at the 0.05 level (2-tailed)

^b^ Correlation is significant at the 0.01 level (2-tailed)

**Table 5:** Unadjusted and adjusted correlation of MALAT1 gene expression in SAT and VAT of the whole population study with energy homeostasis-related genes

Characteristics	lncRNA MALAT1 in SAT		lncRNA MALAT1 in VAT	
	Whole Population		Whole Population	
	Unadjusted Pearson coefficient	Adjusted for age and HOMA-IR Pearson coefficient	Unadjusted Pearson coefficient	Adjusted for age and HOMA-IR Pearson coefficient
PPARγ	0.329^a^	0.299	0.070	0.086
PGC1α	0.153	0.250	0.455^b^	0.424^b^
SREBP	0.640^b^	0.589^b^	0.225	0.193
FAS	0.573^b^	0.513^b^	0.261	0.210
ACC	0.411^b^	0.401^b^	0.186	0.153
LncRNA TUG1	0.299	0.346^a^	− 0.380^a^	− 0.324^a^

SAT, subcutaneous adipose tissue; VAT, visceral adipose tissue; MALAT1, metastasis-associated lung adenocarcinoma transcript 1; TUG1, taurine upregulated gene 1; PPARγ, peroxisome proliferator-activated receptor gamma; PGC1α, PPARγ coactivator-1 alpha; SREBP-1, sterol regulatory element-binding protein α; FAS, fatty acid synthase; ACC, acetyl-CoA carboxylase; HOMA-IR, homeostasis model assessment of insulin resistance

^a^ Correlation is significant at the 0.05 level (2-tailed)

^b^ Correlation is significant at the 0.01 level (2-tailed)

The correlation of transcript levels of TUG1 with the expression of afore-mentioned genes was examined in the whole population using a BMI-adjusted Pearson partial correlation coefficient.

The results revealed that PGC1α (r = 0.375; P = 0.02), SREBP-1c (r = 0.430; P = 0.007), FAS (r = 0.359; P = 0.027), ACC (r = 0.388; P = 0.016), and MALAT1(r = 0.373;P = 0.02) retained significant partial correlation with SAT transcript level of TUG1 among the variables shown in Table 3 after being adjusted for BMI. Moreover, MALAT1 transcript levels(r = − 0.330; P = 0.04) in VAT retained a significant partial correlation with TUG1 gene expression in VAT after being adjusted for BMI.

Positive correlations were also found between MALAT1 gene expression and PPARγ (r = 0.329; P = 0.04), SREBP-1c (r = 0.640; P < 0.0001), FAS (r = 0.573; P < 0.0001), and ACC (r = 0.411; P = 0.009) gene expression in the SAT of all participants. Moreover, there was a positive correlation between the gene expression of MALAT1 and PGC1α in the VAT of the studied individuals (r = 0.455; P = 0.004). In contrast, MALAT1 expression was inversely correlated with TUG1 expression in the VAT (r = − 0.380; P = 0.017)(Table 5). Then, all correlations were adjusted for age and HOMA-IR. The results showed that the correlation of MALAT1 gene expression in SAT with TUG1,SREBP-1c, FAS, and ACC gene expression is independent of age and HOMA-IR. Moreover, MALAT1 gene expression in VAT of all participants showed a statistically significant correlation with PGC-1α and TUG1 of age and HOMA-IR (Table 5).

## Discussion

More recently, researchers have begun studying the potential role of lncRNAs as a novel and potential tool in understanding of the underlying mechanism of obesity [33]. The differential expression of several lncRNAs (e.g. RP11-20G13.3, HOTAIR,GYG2P1, and OLMALINC) has been shown in adipose tissue from both obese and non-obese subjects [14, 34]. Moreover, the contribution of lncRNAs in the field of obesity-related research has been validated in in vitro and animal models more than in specific human tissues [35–37]. Today, little is known about MALAT1 and TUG1 function and regulation in metabolic disorders. To do this, we were the first to evaluate MALAT1 and TUG1 gene expression directly in VAT and SAT of obese and non-obese women, and to study the possible association of transcript levels of these lncRNAs with metabolic profile and expression of several lipogenic and adipogenic genes.

Here we found that the mRNA level of TUG1 was significantly decreased in adipose tissues of obese women, compared to the controls. In line with the present data, Long et al. demonstrated that TUG1 expression was decreased in diabetic animal models, compared to non-diabetic models. A similar pattern was also found in human diabetic kidney samples [30]. On the other hand, TUG1 down-regulation has been observed in insulin resistance, with a primary effect on islet cell apoptosis [9]. Another important finding of the current study is that SAT mRNA levels of TUG1 were positively correlated with SAT mRNA expression of PGC1α, SREBP-1c, FAS, and ACC independent of age and insulin resistance status.

Supporting the present data, previous studies have reported the induction of several lncRNAs in adipose tissue and targeting the main transcription factors of lipogenesis and adipogenesis, including PPARγ and SREBP-1 [11, 37–41]. For instance, knockdown of RP11-20G13.3 significantly decreased expression of markers of adipocyte differentiation: PPARγ, C/EBPα, and adiponectin [14]. Moreover, correlation analysis demonstrated that lncRNA RP11-20G13.3 in adipose tissue from obese and non-obese children was positively associated with obesity indices, circulating levels of insulin, LDL-C,and hs-CRP [14].

The contribution of TUG1 in the field of metabolic abnormalities has been mostly limited to in vitro experiments and animal model studies. The exact mechanisms linking TUG1 and MALAT1 to the pathogenesis of obesity cannot be ascertained according to the present study. However, several possibilities derived from experimental investigations should be considered.

Our finding of an inverse correlation of TUG1 gene expression in SAT with circulating hs-CRP corroborates some evidence for the possible cross-talk between TUG1 and inflammation. For example, LPS and TNF-α-induced inflammation was shown to reduce TUG1 expression. Moreover, TUG1 suppression augmented pro-inflammatory cytokine production [42, 43]. Hence, it is tempting to speculate that the decreased expression of TUG1 in adipose tissue may be partly related to the inflammatory milieu in obesity which in turn exacerbates inflammation in obesity, reflected by high levels of hs-CRP. However, the involvement of other unknown mechanisms should not be ruled out.

A recent study demonstrated that TUG1 inhibited extracellular matrix (ECM) accumulation through post-transcriptional modification of PPARγ, which suggests a new insight for diabetic nephropathy [28]. There is also evidence that TUG1 through a PGC1α-dependent mechanism, contributes to diabetic kidney disease development [17, 30]. Moreover, overexpression of TUG1 in an animal model of diabetic nephropathy inhibited diabetes-induced ROS formation and albuminuria [44]. Based on the observations mentioned above and the positive correlation of TUG1 expression with PGC1α in our correlation analysis, it is tempting to speculate that a decrease in the TUG1 expression in obese women may contribute to kidney diseases in obese patients. To support this notion, a statistically significant correlation was found between TUG1 expression and creatinine and eGFR, as the main renal function index.

Considering the well-established role of energy imbalance and dysregulation of lipid metabolism in obesity pathogenesis [45, 46] which was reflected by a change in the expression of relevant genes (PPARγ,PGC1α, FAS, and ACC), it seems that the reduced expression of TUG1 in adipose tissue plays a part in the initiation and development of obesity through affecting key adipogenic and lipogenic genes. However, mechanistic studies need to expand current knowledge about the biological roles of lncRNAs in the regulation of lipogenic and adipogenic markers.

In the present study, no statistically significant difference was observed in MALAT1 mRNA levels in both VAT and SAT between the two study groups. In contrast with the present results, MALAT1 was seen to be significantly down-regulated in SAT from both genetic and diet-induced models of obesity. Moreover, a decrease in MALAT1 expression was observed in VAT from old male C57BL/6 J mice and also from old men [23].

The literature on the role of MALAT1 in obesity and related disorders is scarce and controversial. MALAT1 was recently shown to be decreased in white adipose tissue from obese mice, however, its deletion had no effects (either stimulatory or inhibitory) on the diet-induced gain in adipose tissue and lipid homeostasis of obese mice [23]. In contrast, Patel et al. showed that MALAT1 expression is significantly higher in medium secreted from adipose-derived stem cells of obese subjects [24]. Previous studies have indicated a higher expression of MALAT1 in different models of diabetes and NAFLD, which was associated with oxidative stress induction and inflammatory factor production [47–49].

The role of MALAT1 was demonstrated in several metabolic disorders, including hepatic steatosis and IR, by the stabilization of nuclear SREBP-1c through inhibiting its ubiquitination. In addition, MALAT1 knockdown caused a decrease in the expression of FAS in HepG2 cell and primary mouse hepatocytes, which consequently reduced lipid accumulation. However, palmitate-induced MALAT1, along with SREBP-1c overexpression, increased hepatic lipid accumulation in hepatic cells [29, 41]. Concordant with this evidence, we found that MALAT1 mRNA expression was positively correlated with SAT expression levels of SREBP-1c, PPARγ, and their targets, FAS and ACC, and also with VAT mRNA levels of PGC1α.

There is also a report that MALAT1 ablation suppressed abnormal ROS production and improved insulin secretion in response to glucose challenge in male mice [50]. In agreement with this notion, we observed that MALAT1 gene expression in SAT was positively correlated with HOMA-IR in all participants. Although we found no significant difference in MALAT1 gene expression, it seems likely that alteration in MALAT1 transcript may contribute to IR susceptibility in the context of obesity possibly through changes in some lipid-homeostasis-related genes in fat depots.

It is worth bearing in mind that many lncRNAs have been identified in mammalian genomes, but the majority of lncRNAs lack a distinct function [51]. However, the present study, along with others, can provide partial evidence for the role of MALAT1 and TUG1 in the regulation of lipid hemostasis in adipose tissue from obese subjects.

Although to the best of the authors’ knowledge, this study was one of the first to draw attention to the possible role of MALAT1 and TUG1 in the pathogenesis of obesity in humans, some limitations of the current study deserve to be mentioned. Firstly, the study had a cross-sectional design, which precludes the deduction of any causal relationship between lncRNAs gene expression and obesity indices. Secondly, epigenetic factors such as lncRNAs are influenced by environmental factors including physical activity and nutrient status. Therefore, the absence of these data can be considered as a limitation to in-depth interpret our results. Thirdly, since gender influences epigenetic factors, the presented data is only applicable for women and it must be replicated in men. Thus, another point which warrants consideration is the need for more clinical studies involving much larger samples and examining both men and women. Furthermore, considering that adipokines, especially leptin and adiponectin have potential roles in the pathogenesis of obesity, evaluation of association between these adipokines with two studied lncRNAs is suggested.

## Conclusions

Collectively, we observed a lower expression of TUG1 in obese women and its inverse correlation with obesity indices, hs-CRP, and creatinine levels. In addition, a positive correlation of MALAT1 gene expression and HOMA-IR was seen in the SAT of all participants. It might suggest the contribution of these lncRNAs in the pathogenesis of obesity. More importantly, MALAT1 and TUG1 transcript levels showed positive correlations with master lipogenic and adipogenic genes which might be indicative of the possible role of MALAT1 and TUG1 in obesity possibly through regulating the lipogenic and adipogenic genes. Although this study cannot absolutely confirm the association between MALAT1 and TUG1 and obesity, it can add to the literature on the possible role of afore-mentioned lncRNAs in human obesity. These findings may have major implications for controlling this disease, which necessitate further investigation.

## Supplementary information

**Additional file 1: Table S1.** Forward and reverse primers used for real-time PCR.

## Data Availability

The datasets used and/or analysed during the current study are available from the corresponding author on reasonable request.

## References

[1] Friedman JM (2000). Obesity in the new millennium. Nature.

[2] Wei S, Du M, Jiang Z, Hausman GJ, Zhang L, Dodson MV (2016). Long noncoding RNAs in regulating adipogenesis: new RNAs shed lights on obesity. Cell Mol Life Sci.

[3] Sam S, Mazzone T (2014). Adipose tissue changes in obesity and the impact on metabolic function. Transl Res..

[4] Greenberg AS, Obin MS (2006). Obesity and the role of adipose tissue in inflammation and metabolism. Am J Clin Nutr.

[5] Ibrahim MM (2010). Subcutaneous and visceral adipose tissue: structural and functional differences. Obes Rev.

[6] Ransohoff JD, Wei Y, Khavari PA (2018). The functions and unique features of long intergenic non-coding RNA. Nat Rev Mol Cell Biol.

[7] Lee JT (2009). Lessons from X-chromosome inactivation: long ncRNA as guides and tethers to the epigenome. Genes Dev.

[8] Flynn RA, Chang HY (2014). Long noncoding RNAs in cell-fate programming and reprogramming. Cell Stem Cell.

[9] Yin DD, Zhang EB, You LH, Wang N, Wang LT, Jin FY (2015). Downregulation of lncRNA TUG1 affects apoptosis and insulin secretion in mouse pancreatic beta cells. Cell Physiol Biochem.

[10] Chen G, Yu D, Nian X, Liu J, Koenig RJ, Xu B (2016). LncRNA SRA promotes hepatic steatosis through repressing the expression of adipose triglyceride lipase (ATGL). Sci Rep..

[11] Chen J, Liu Y, Lu S, Yin L, Zong C, Cui S (2017). The role and possible mechanism of lncRNA U90926 in modulating 3T3-L1 preadipocyte differentiation. Int J Obes (Lond)..

[12] Dallner OS, Marinis JM, Lu YH, Birsoy K, Werner E, Fayzikhodjaeva G (2019). Dysregulation of a long noncoding RNA reduces leptin leading to a leptin-responsive form of obesity. Nat Med.

[13] Gao H, Kerr A, Jiao H, Hon CC, Ryden M, Dahlman I (2018). Long non-coding RNAs associated with metabolic traits in human white adipose tissue. EBioMedicine..

[14] Liu Y, Ji Y, Li M, Wang M, Yi X, Yin C (2018). Integrated analysis of long noncoding RNA and mRNA expression profile in children with obesity by microarray analysis. Sci Rep..

[15] Fajas L, Schoonjans K, Gelman L, Kim JB, Najib J, Martin G (1999). Regulation of peroxisome proliferator-activated receptor gamma expression by adipocyte differentiation and determination factor 1/sterol regulatory element binding protein 1: implications for adipocyte differentiation and metabolism. Mol Cell Biol.

[16] Kersten S (2001). Mechanisms of nutritional and hormonal regulation of lipogenesis. EMBO Rep.

[17] Beaven SW, Matveyenko A, Wroblewski K, Chao L, Wilpitz D, Hsu TW (2013). Reciprocal regulation of hepatic and adipose lipogenesis by liver X receptors in obesity and insulin resistance. Cell Metab.

[18] Knight BL, Hebbachi A, Hauton D, Brown AM, Wiggins D, Patel DD (2005). A role for PPARalpha in the control of SREBP activity and lipid synthesis in the liver. Biochem J..

[19] Xu X, So JS, Park JG, Lee AH (2013). Transcriptional control of hepatic lipid metabolism by SREBP and ChREBP. Semin Liver Dis.

[20] Gavrilova O, Haluzik M, Matsusue K, Cutson JJ, Johnson L, Dietz KR (2003). Liver peroxisome proliferator-activated receptor gamma contributes to hepatic steatosis, triglyceride clearance, and regulation of body fat mass. J Biol Chem.

[21] Ji P, Diederichs S, Wang W, Boing S, Metzger R, Schneider PM (2003). MALAT-1, a novel noncoding RNA, and thymosin beta4 predict metastasis and survival in early-stage non-small cell lung cancer. Oncogene.

[22] Szymanski M, Barciszewska MZ, Erdmann VA, Barciszewski J (2005). A new frontier for molecular medicine: noncoding RNAs. Biochim Biophys Acta.

[23] Carter S, Miard S, Boivin L, Sallé-Lefort S, Picard F (2018). Loss of Malat1 does not modify age- or diet-induced adipose tissue accretion and insulin resistance in mice. PLoS ONE.

[24] Patel RS, Carter G, El Bassit G, Patel AA, Cooper DR, Murr M (2016). Adipose-derived stem cells from lean and obese humans show depot specific differences in their stem cell markers, exosome contents and senescence: role of protein kinase C delta (PKCdelta) in adipose stem cell niche. Stem Cell Investig..

[25] Sun Q, Xu H, Xue J, Yang Q, Chen C, Yang P (2018). MALAT1 via microRNA-17 regulation of insulin transcription is involved in the dysfunction of pancreatic beta-cells induced by cigarette smoke extract. J Cell Physiol.

[26] Young TL, Matsuda T, Cepko CL (2005). The noncoding RNA taurine upregulated gene 1 is required for differentiation of the murine retina. Curr Biol.

[27] Smolle M, Uranitsch S, Gerger A, Pichler M, Haybaeck J (2014). Current status of long non-coding RNAs in human cancer with specific focus on colorectal cancer. Int J Mol Sci.

[28] Duan LJ, Ding M, Hou LJ, Cui YT, Li CJ, Yu DM (2017). Long noncoding RNA TUG1 alleviates extracellular matrix accumulation via mediating microRNA-377 targeting of PPARgamma in diabetic nephropathy. Biochem Biophys Res Commun.

[29] Yan C, Chen J, Chen N (2016). Long noncoding RNA MALAT1 promotes hepatic steatosis and insulin resistance by increasing nuclear SREBP-1c protein stability. Sci Rep..

[30] Long J, Badal SS, Ye Z, Wang Y, Ayanga BA, Galvan DL (2016). Long noncoding RNA Tug1 regulates mitochondrial bioenergetics in diabetic nephropathy. J Clin Invest..

[31] Saleem M, Florkowski CM, George PM, Woltersdorf WW (2006). Comparison of two prediction equations with radionuclide glomerular filtration rate: validation in routine use. Ann Clin Biochem.

[32] Bustin SA, Benes V, Garson JA, Hellemans J, Huggett J, Kubista M (2009). The MIQE guidelines: minimum information for publication of quantitative real-time PCR experiments. Clin Chem.

[33] Latorre J, Fernández-Real JM (2018). LncRNAs in adipose tissue from obese and insulin-resistant subjects: new targets for therapy?. EBioMedicine..

[34] Divoux A, Karastergiou K, Xie H, Guo W, Perera RJ, Fried SK (2014). Identification of a novel lncRNA in gluteal adipose tissue and evidence for its positive effect on preadipocyte differentiation. Obesity (Silver Spring)..

[35] Yu L, Tai L, Zhang L, Chu Y, Li Y, Zhou L (2017). Comparative analyses of long non-coding RNA in lean and obese pig. Oncotarget..

[36] Lo KA, Huang S, Walet ACE, Zhang ZC, Leow MK, Liu M (2018). Adipocyte long-noncoding RNA transcriptome analysis of obese mice identified Lnc-Leptin, which regulates leptin. Diabetes..

[37] Huang Y, Jin C, Zheng Y, Li X, Zhang S, Zhang Y (2017). Knockdown of lncRNA MIR31HG inhibits adipocyte differentiation of human adipose-derived stem cells via histone modification of FABP4. Sci Rep..

[38] Sun L, Goff LA, Trapnell C, Alexander R, Lo KA, Hacisuleyman E (2013). Long noncoding RNAs regulate adipogenesis. Proc Natl Acad Sci USA..

[39] Huang J, Chen S, Cai D, Bian D, Wang F (2018). Long noncoding RNA lncARSR promotes hepatic cholesterol biosynthesis via modulating Akt/SREBP-2/HMGCR pathway. Life Sci.

[40] Giroud M, Scheideler M (2017). Long non-coding RNAs in metabolic organs and energy homeostasis. Int J Mol Sci..

[41] van Solingen C, Scacalossi KR, Moore KJ (2018). Long noncoding RNAs in lipid metabolism. Curr Opin Lipidol.

[42] Zhang H, Li H, Ge A, Guo E, Liu S, Zhang L (2018). Long non-coding RNA TUG1 inhibits apoptosis and inflammatory response in LPS-treated H9c2 cells by down-regulation of miR-29b. Biomed Pharmacother.

[43] Zhao K, Tan JY, Mao QD, Ren KY, He BG, Zhang CP (2019). Overexpression of long non-coding RNA TUG1 alleviates TNF-alpha-induced inflammatory injury in interstitial cells of Cajal. Eur Rev Med Pharmacol Sci..

[44] Li SY, Susztak K (2016). The long noncoding RNA Tug1 connects metabolic changes with kidney disease in podocytes. J Clin Invest..

[45] Klop B, Elte JW, Cabezas MC (2013). Dyslipidemia in obesity: mechanisms and potential targets. Nutrients..

[46] Franssen R, Monajemi H, Stroes ES, Kastelein JJ (2011). Obesity and dyslipidemia. Med Clin North Am.

[47] Yan B, Tao ZF, Li XM, Zhang H, Yao J, Jiang Q (2014). Aberrant expression of long noncoding RNAs in early diabetic retinopathy. Invest Ophthalmol Vis Sci.

[48] Puthanveetil P, Chen S, Feng B, Gautam A, Chakrabarti S (2015). Long non-coding RNA MALAT1 regulates hyperglycaemia induced inflammatory process in the endothelial cells. J Cell Mol Med.

[49] Leti F, Legendre C, Still CD, Chu X, Petrick A, Gerhard GS (2017). Altered expression of MALAT1 lncRNA in nonalcoholic steatohepatitis fibrosis regulates CXCL5 in hepatic stellate cells. Transl Res..

[50] Chen J, Ke S, Zhong L, Wu J, Tseng A, Morpurgo B (2018). Long noncoding RNA MALAT1 regulates generation of reactive oxygen species and the insulin responses in male mice. Biochem Pharmacol.

[51] Kung JT, Colognori D, Lee JT (2013). Long noncoding RNAs: past, present, and future. Genetics.

