# Supplementary material for: Adipose tissue gene expression of long non-coding RNAs; MALAT1, TUG1 in obesity: is it associated with metabolic profile and lipid homeostasis-related genes expression?
Source: Diabetol Metab Syndr. 2020 Apr 29;12:36. doi: 10.1186/s13098-020-00544-0 (PMC7191796; doi:10.1186/s13098-020-00544-0)
Supplement: Supplementary file 1 — Additional file 1: Table S1. Forward and reverse primers used for real-time PCR. [file 13098_2020_544_MOESM1_ESM.docx]

| **Table S1: Forward and reverse primers used for real-time PCR.** | | |
| --- | --- | --- |
| Primer | Forward sequence | Reverse sequence |
| MALAT1 | 5'-GACGAGTTGTGCTGCTATCTT-3' | 5'-GATTCTGTGTTATGCCTGGTTAG-3' |
| TUG1 | 5'-TAGCAGTTCCCCAATCCTTG-3' | 5'-CACAAATTCCCATCATTCCC-3' |
| PPARγ | 5'-GAGTACCAAAGTGCAATCAAAG-3' | 5'-CTCCGGAAGAAACCCTTGCATC-3' |
| PGC1α | 5'-CACAACACTTACAAGCCAAACCA-3' | 5'-CAGTTCCAGAGAGTTCCACACTT-3' |
| SREBP-1c | 5'-GGATGGTGTTCACTCGGTA-3' | 5'-GGTGATATGTGTCTGCGTC-3' |
| FAS | 5'-GAGGAAGGAGGGTGTGTTT-3' | 5'-CGGGGATAGAGGTGCTGA-3' |
| ACC | 5'-TGAGGACAGCAAGGCAAG-3' | 5'-CAGGACAGGCAGAGGAAGA-3' |
| GAPDH | 5'-TGCACCACCAACTGCTTAGC-3' | 5'-GGCATGGACTGTGGTCATGAG-3' |
| β-actin | 5'-TCCTTCCTGGGCATGGAGT-3' | 5'-ACTGTGTTGGCGTACAGGTC-3' |
| MALAT1, metastasis-associated lung adenocarcinoma transcript 1; TUG1, taurine upregulated gene 1; PPARγ, peroxisome proliferator-activated receptor gamma; PGC1α, PPARγ coactivator-1 alpha; SREBP-1c, sterol regulatory element-binding protein 1c; FAS, fatty acid synthase; ACC, acetyl-CoA carboxylase; GAPDH, glyceraldehyde 3-phosphate dehydrogenase. | | |
